# The Changes in Bioactive Compounds and Antioxidant Activity of Chia (*Salvia hispanica* L.) Herb under Storage and Different Drying Conditions: A Comparison with Other Species of Sage

**DOI:** 10.3390/molecules27051569

**Published:** 2022-02-26

**Authors:** Kinga Dziadek, Aneta Kopeć, Michał Dziadek, Urszula Sadowska, Katarzyna Cholewa-Kowalska

**Affiliations:** 1 Department of Human Nutrition and Dietetics, Faculty of Food Technology, University of Agriculture in Krakow, 21 Mickiewicza Ave., 31-120 Krakow, Poland; aneta.kopec@urk.edu.pl; 2 Department of Inorganic Chemistry, Faculty of Chemistry, Jagiellonian University, 2 Gronostajowa St., 30-387 Krakow, Poland; michal.dziadek@uj.edu.pl or; 3 Department of Glass Technology and Amorphous Coatings, Faculty of Materials Science and Ceramics, AGH University of Science and Technology, 30 Mickiewicza Ave., 30-059 Krakow, Poland; cholewa@agh.edu.pl; 4 Institute of Machinery Exploitation, Ergonomics and Production Processes, Faculty of Production and Power Engineering, University of Agriculture in Krakow, 21 Mickiewicza Ave., 31-120 Krakow, Poland; urszula.sadowska@urk.edu.pl

**Keywords:** sage, *Salvia hispanica* herb, chia herb, drying, freeze-drying, storage, polyphenols

## Abstract

Studies on herb chia (*Salvia hispanica* L.) are very limited. Therefore, the aim of this study was to assess how different drying methods and periods of storage affect the bioactive properties of the herb *Salvia hispanica* and to compare it with other species of sage (*Salvia officinalis* L. and *Salvia sclarea* L.). In fresh herbs, directly after drying (freeze-drying, natural drying, and drying at 30, 40, and 50 °C), and after storage (3, 6, and 12 months), the following analyses were performed: content of total carotenoids and total polyphenols, polyphenol profile (including 25 compounds), and antioxidant activity. Additionally, the basic chemical compositions of the herbs were analyzed. To the best of our knowledge, the content of total carotenoids and the quantitative polyphenol profile in *Salvia hispanica* and *Salvia sclarea* were evaluated for the first time. The obtained results showed that the barely investigated herb *Salvia hispanica* is rich in polyphenolic compounds and shows high antioxidant activity. In all the tested species, rosmarinic acid was the most abundant polyphenolic compound. The use of different drying methods allowed us to determine that freeze-drying was the most effective for preserving polyphenols and carotenoids. Long-term storage up to 12 months resulted in a gradual reduction in antioxidant activity and in the content of polyphenols and carotenoids.

## 1. Introduction

Herbs have been used for centuries as natural remedies to fight some diseases. With the development of synthetic drugs in the early 20th century, the use of herbs in medicine declined significantly. However, in recent years, there has been growing interest in folk medicine involving medical plants and herbs [1,2,3].

One group of beneficial medicinal plants is the genus *Salvia*, which belongs to the *Lamiaceae* family and includes approximately 900 species [4]. It is cultivated throughout the world for use in food industries as spices (to flavor meats such as pork, sausage, and poultry) as well as in cosmetics [5,6], newly formulated biomaterials [7,8,9], and active food packaging [10]. Studies have indicated that several species of sage (including *Salvia officinalis* L. and *Salvia sclarea* L.) show biological activities such as antioxidant, antibacterial, antifungal, anticancer, antimutagenic, anti-inflammatory, and antidepressant activities [5,6,11,12,13,14,15,16]. These beneficial effects result from numerous compounds belonging to different chemical groups, mainly polyphenolic compounds (phenolic acids and flavonoids), carotenoids, and essential oils [12,17].

Fresh herbs are treated using various preservation processes, to extend their shelf life while maintaining the highest quality. To protect their microbiological stability and biological activities, the level of water must be decreased below 10% [18]. Drying is one of the most commonly used methods of preservation. It reduces microbial growth, and therefore allows for long-term storage. Furthermore, during the drying process some biochemical changes occur that can improve the quality. Moreover, freeze-drying is an increasingly used drying method in the food industry that preserves the properties of the fresh plant (including chemical composition and biological activities) to the greatest extent. Additionally, these treatments allow for a decrease in the cost of transport and storage by reducing the product weight [19,20,21]. Nowadays, innovative drying technologies such as the reaction engineering approach (REA), as well as microwave, infrared, ultrasonic, low-pressure superheated steam, and pulse combustion spray drying are under investigation. These methods are promising prospects for the food industry. They can enhance the quality of the product, as well as improve the drying efficiency, reducing energy consumption and environmental impact [22,23].

*Salvia hispanica* L. (known as chia) is cultivated mainly for its seeds. The growing interest in chia in recent years has been due to its beneficial fatty acid profile. Chia seeds contain highly unsaturated fatty acids, mainly linoleic and α-linolenic acid [24,25]. They are a source of protein, with a proper balance of the essential amino acids, especially methionine and cysteine [26]. Chia seeds also contain bioactive compounds, mainly dietary fiber but also polyphenolic compounds such as myricetin, quercetin, kaempferol, and chlorogenic acids [25,27,28]. However, there are limited studies on the herb of *Salvia hispanica* [27,29].

The aim of this study was to assess how different drying methods and periods of storage affect the content of bioactive compounds (including the polyphenol profile) and antioxidant activity of the herb *Salvia hispanica*, as well as to compare it with other species of sage (*Salvia officinalis* and *Salvia sclarea*). To the best of our knowledge, the content of total carotenoids and the quantitative polyphenol profile in *Salvia hispanica* and *Salvia sclarea* were evaluated for the first time in this study.

## 2. Materials and Methods

### 2.1. Plant Material

The following species of sage were analyzed: *Salvia hispanica* (chia), *Salvia officinalis*, and *Salvia sclarea*. All species were grown in the same location (Bielsko-Biała, Silesia, Poland) and under the same conditions. No pesticides and no hydration were used during cultivation. The herb samples were randomly collected from plants. The samples were dried using the following methods: freeze-drying, natural drying, and drying at 30 °C, 40 °C, and 50 °C. Lyophilization was conducted in a vacuum freeze dryer (Alpha 1-4 LSCplus, Martin Christ Gefriertrocknungsanlagen GmbH, Osterode am Harz, Germany). Natural drying was performed in a dry and shaded room, while convection drying at temperatures of 30 °C, 40 °C, and 50 °C was conducted in a laboratory dryer. The volume of the dryer working chamber was 100 dm^3^. Natural drying and drying at elevated temperatures were carried out until a water content of 100 mL∙kg^−^^1^ was achieved. Additionally, after drying, herbs were tightly packed and stored for 3, 6, and 12 months at room temperature, protected from sunlight. Fresh and dried herbs, before and after the different storage periods, were used to prepare methanolic extracts and to evaluate the total carotenoids content. Additionally, the freeze-dried herbs were used to determine the basic chemical composition.

### 2.2. Basic Chemical Composition

In the freeze-dried samples, the contents of total protein (procedure no. 950.36), crude fat (procedure no. 935.38), total dietary fiber (procedure no. 991.43) and ash (procedure no. 930.05) were determined according to the AOAC [30] methods. The content of digestible carbohydrates was calculated using the following equation [31]:

digestible  carbohydrates=100−(protein+crude fat+ash+dietary fiber)

### 2.3. Determination of Total Carotenoids

The content of total carotenoids was determined by extracting carotenoids from the samples using an acetone–hexane mixture (4:6 *v*/*v*) (Chempur, Piekary Śląskie, Poland), according to the Polish standard with some modifications [32]. Samples containing 1 g of fresh or 0.2 g of dried sage herbs were weighed into a porcelain mortar. Thereafter, approximately 0.3 g of sand was used to extract the dye using an acetone–hexane mixture. The extracts were poured into a test cylinder. Then, the extract volume was read. The extracts were shaken and left in a dark place for 30 min. The absorbance was measured at 450 nm using a spectrophotometer (UV-1800, Rayleigh, Beijing Beifen-Ruili Analytical Instrument Co., Ltd., Beijing, China). Results were calculated based on the β-carotene (Sigma-Aldrich, St. Louis, MO, USA) calibration curve.

### 2.4. Preparation of the Extracts

The content of total polyphenols, the polyphenol profile, and the antioxidant activity were determined in methanolic extracts. To prepare the extracts, 0.3 g of fresh or 0.2 g of dried herbs, were used, with 60 mL of 0.1% formic acid in 70% methanol (*v*/*v*) (POCH, Gliwice, Poland). The extracts were prepared by shaking for 2 h at room temperature (water bath shaker type 357, Elpan, Lubawa, Poland). Then the samples were filtered using filter paper. The filtrates were stored at a temperature of −20 °C for further analysis.

### 2.5. Determination of Total Polyphenols

The level of total polyphenols was estimated using Folin–Ciocalteu reagent (Sigma-Aldrich, Saint-Louis, MO, USA), as previously described [33]. Results were expressed as mg of gallic acid (GA) per 100 g DW (dry weight) of the sample.

### 2.6. Determination of Polyphenol Profile

High-performance liquid chromatography (HPLC) was used to evaluate the polyphenol profile. The analysis was conducted using a Prominence-i LC-2030C 3D Plus system (Shimadzu, Kyoto, Japan) equipped with a diode array detector (DAD). The separation was performed on a Luna Omega 5 µm Polar C18, 100 A, 250 × 10 mm column (Phenomenex, CA, USA) at 40 °C. The mobile phase was a mixture of two eluents: A—0.1% formic acid in water (*v*/*v*) and B—0.1% formic acid in methanol (*v*/*v*). The flow rate of the mobile phase was 1.2 mL min^−^^1^. The analysis was carried out with the following gradient conditions: from 20% to 40% B in 10 min, 40% B for 10 min, from 40% to 50% B in 10 min, from 50% to 60% B in 5 min, 60% B for 5 min, from 60 to 70% B in 5 min, from 70% to 90% B in 5 min, 90% B for 5 min, from 90% to 20% B (the initial condition) in 1 min, and 20% B for 4 min, resulting in a total run time of 60 min. The injection volume was 20 μL.

The quantification of individual polyphenols in methanolic extracts was performed by establishing calibration curves using the standards. The calibration curves for the standards were linear with R^2^ > 0.995.

The detection of 4-hydroxybenzoic acid, myricetin, quercetin, luteolin, and isorhamnetin was performed at 254 nm, rutin at 256 nm, vanillic acid at 260 nm, kaempferol at 264 nm, apigenin and acacetin at 267 nm, gallic acid at 271 nm, hispidulin at 273 nm, syringic acid at 274 nm, catechin and epicatechin at 278 nm, naringin and carnosol at 283 nm, hesperidin and carnosic acid at 284 nm, p-coumaric acid at 310 nm, caffeic acid, ferulic acid, and sinapinic acid at 323 nm, chlorogenic acid at 326 nm, and rosmarinic acid at 329 nm. The data were integrated and analyzed using the LabSolutions software (Shimadzu, Kyoto, Japan).

### 2.7. Determination of Antioxidant Activity

The antioxidant activity was determined using the ABTS method (2,2′-azino-*bis*(3-ethylbenzthiazoline-6-sulfonic acid)), as previously reported [34]. The obtained results were expressed as micromoles of Trolox equivalent per gram (DW) of the sample.

### 2.8. Statistical Analysis

Statistical analysis of the obtained data was performed using the program Statistica version 13.1, Dell Inc., Tulsa, OK, USA, 2016. The results were analyzed using one-way analysis of variance (ANOVA). Significant differences between mean values (*n* = 3) were compared using Duncan’s test.

## 3. Results

### 3.1. Basic Chemical Composition

*Salvia sclarea* was characterized by significantly the highest level of ash (18.39 g∙100 g^−1^ DW) and protein (14.49 g∙100 g^−1^ DW) (Table 1). The highest amount of crude fat was determined in *Salvia officinalis* (4.06 g∙100 g^−1^ DW), while the lowest was found in *Salvia hispanica* (1.67 g∙100 g^−1^ DW) herb. The highest content of dietary fiber (52.98 g∙100 g^−1^ DW) and, at the same time, the lowest level of digestible carbohydrates (24.82 g∙100 g^−1^ DW) were measured in *Salvia officinalis*, in comparison to the other species.

### 3.2. The Content of Total Carotenoids

Among the fresh herbs, significantly the highest content of total carotenoids was found in *Salvia* *sclarea* (109.06 mg∙100 g^−1^ DW) (Table 2). The drying methods and storage significantly affected the content of these compounds (Table 3). The freeze-dried herbs showed the highest content of carotenoids (71.04 mg∙100 g^−1^ DW in *Salvia sclarea*, 64.87 mg∙100 g^−1^ DW in *Salvia hispanica*, and 57.98 mg∙100 g^−1^ DW in *Salvia officinalis*), indicating that this method caused the smallest loss in comparison to other drying methods. On the other hand, samples dried at 30 °C were characterized by the lowest level of these compounds (42.24 mg∙100 g^−1^ DW in *Salvia sclarea*, 27.93 mg∙100 g^−1^ DW in *Salvia hispanica*, and 23.60 mg∙100 g^−1^ DW in *Salvia officinalis*), resulting from the highest losses. Storage for 3, 6, and 12 months caused an increasing loss in carotenoid content in all tested species of sage. The highest loss was observed after 12 months of storage. The loss of carotenoids in the studied samples during storage seemed not to depend on the drying method.

### 3.3. The Content of Total Polyphenols

In the fresh herbs, *Salvia hispanica* and *Salvia officinalis* were characterized by a significantly higher content of total polyphenols (9.76 g∙100 g^−1^ DW and 9.47 g∙100 g^−1^ DW, respectively) in comparison to *Salvia sclarea* (4.95 g∙100 g^−1^ DW) (Table 2). The drying method affected the level of these compounds significantly (Table 4). In the herbs *Salvia hispanica* and *Salvia officinalis* , an increase in the content of polyphenols was found in the samples after freeze-drying and natural drying. However, the highest content was observed after freeze-drying (12.89 g∙100 g^−1^ DW in *Salvia officinalis* and 11.84 g∙100 g^−1^ DW in *Salvia hispanica*). In these herbs, a loss in polyphenolic compounds was found in samples after drying at 30 °C, 40 °C, and 50 °C. The lowest level of these compounds was determined in these herbs after drying at 50 °C (6.11 g∙100 g^−1^ DW in *Salvia officinalis* and 5.49 g∙100 g^−1^ DW in *Salvia hispanica*). However, for the *Salvia sclarea* samples, all drying methods caused an increase in the content of polyphenols. The greatest changes were found after natural drying (8.52 g∙100 g^−1^ DW). Storage resulted in a decrease in levels of polyphenolic compounds in all the tested samples. The lowest levels of these compounds, caused by the highest loss, were found after 12 months of storage.

### 3.4. Polyphenol Profile

The following polyphenols were found: 4-hydroxybenzoic acid, caffeic acid, chlorogenic acid, ferulic acid, gallic acid, p-coumaric acid, rosmarinic acid, sinapinic acid, syringic acid, vanillic acid, acacetin, apigenin, catechin, epicatechin, hesperidin, hispidulin, isorhamnetin, kaempferol, luteolin, myricetin, naringin, quercetin, rutin, carnosic acid, and carnosol. The polyphenols detected in *Salvia hispanica*, *Salvia officinalis,* and *Salvia sclarea*, divided into phenolic acids, flavonoids, and phenolic diterpenes, are shown in Table 5, Table 6, and Table 7, respectively.

In the fresh herb of *Salvia hispanica*, 4-hydroxybenzoic acid, ferulic acid, syringic acid, acacetin, catechin, epicatechin, hispidulin, kaempferol, and quercetin were not identified. Rosmarinic acid (1358.80 mg∙100 g^−1^ DW), sinapinic acid (196.71 mg∙100 g^−1^ DW), naringin (199.07 mg∙100 g^−1^ DW), and rutin (197.59 mg∙100 g^−1^ DW) were the dominant polyphenols in this species. In the fresh herb of *Salvia officinalis*, apigenin, catechin, and hispidulin were not detected. However, the highest contents of rosmarinic acid (1488.28 mg∙100 g^−1^ DW), hesperidin (346.43 mg∙100 g^−1^ DW), naringin (523.85 mg∙100 g^−1^ DW), and rutin (317.77 mg∙100 g^−1^ DW) were measured. In the fresh herb of *Salvia sclarea*, p-coumaric acid, acacetin, catechin, hispidulin, kaempferol, myricetin, and quercetin were not found. The following polyphenolic compounds were dominant in this species of sage: rosmarinic acid (346.32 mg∙100 g^−1^ DW), rutin (127.41 mg∙100 g^−1^ DW), and carnosol (296.72 mg∙100 g^−1^ DW).

The results indicated that the method of drying significantly affected the level of individual polyphenolic compounds. Furthermore, differences in the polyphenol profiles before and after drying were found. In the herb of *Salvia hispanica* after drying, 4-hydroxybenzoic acid, ferulic acid, syringic acid, epicatechin, hispidulin, kaempferol, and quercetin were additionally determined. On the other hand, gallic acid was detected only in the fresh sample, after freeze-drying, and after natural drying. In samples of *Salvia officinalis* after drying, apigenin (with the exception of the sample after natural drying), catechin, and hispidulin were additionally identified. However, in the dried herb of *Salvia sclarea*, p-coumaric acid, acacetin, kaempferol, myricetin, and quercetin were detected (the exceptions were p-coumaric acid, kaempferol, and quercetin in the sample after natural drying).

In all samples, storage resulted in a decrease in the content of particular polyphenolic compounds. However, the rates of loss during storage were different for individual compounds.

### 3.5. Antioxidant Activity

The results indicated that the fresh herbs *Salvia hispanica* and *Salvia officinalis* were characterized by higher antioxidant activity (713.26 µmol Trolox∙g^−1^ DW and 651.48 µmol Trolox∙g^−1^ DW, respectively) than *Salvia sclarea* (568.49 µmol Trolox∙g^−1^ DW) (Table 2). In the herbs *Salvia hispanica* and *Salvia officinalis* after freeze-drying, natural drying, and drying at 30 °C and 40 °C, an increase in the ability to scavenge free radicals (highest in the samples after freeze-drying and natural drying) was observed (Table 8). In these groups of samples, drying at 50 °C caused a decrease in the antioxidant activity. In the herb *Salvia sclarea* the highest increase in antioxidant capacity was found in the sample after natural drying, and the lowest was measured after drying at 50 °C. After 3, 6, and 12 months of storage, a decrease in the ability to scavenge free radicals was observed.

## 4. Discussion

As there are only very limited studies on the herb *Salvia hispanica*, the basic chemical composition, bioactive compounds, and antioxidant activity were analyzed and compared to other known species of sage (*Salvia officinalis* and *Salvia sclarea*). As drying is one of the most commonly used methods for herb preservation and shelf-life extension, the effects of different drying methods and periods of storage on the bioactive properties of the analyzed species of sage were evaluated.

In the tested samples, dietary fiber was the most abundant ingredient and fat was the least abundant. In the available literature, there are limited data regarding the basic chemical composition of sage. Peiretti and Gai [29], who studied *Salvia hispanica*, reported a lower content of ash (7.7 g·100 g^−^^1^ DW) and protein (5.7 g·100 g^−^^1^ DW) compared to our results. Other results indicated that different herbs from *Lamiaceae* family were also characterized by a lower content of ash, protein, carbohydrates, and dietary fiber [35,36,37].

The content of total carotenoids in fresh *Salvia officinalis* was 63.48 mg·100 g^−^^1^ DW. Daly et al. [38], Cvitković et al. [39], and Murkovic et al. [40], who studied commonly consumed herbs, reported a lower level of total carotenoids in sage. However, Martins et al. [41] showed higher values for *Salvia officinalis* leaves, compared to our results obtained for the whole herb (leaves and stems). This suggests that carotenoids are mainly accumulated in the leaves of the herb. This was confirmed also on the basis of research on different herbs [42]. To the best of our knowledge, the content of total carotenoids in *Salvia hispanica* and *Salvia sclarea* herb has not previously been determined.

Carotenoids are thermally and oxidatively labile compounds. They contain a chromophore group, which consists of seven or more conjugated double bonds. This structure allows the compounds to absorb visible light, giving them the appropriate color. This system is also responsible for the high thermal and oxidative sensitivity [43]. The obtained results showed that all the drying methods used caused a decrease in the carotenoids content in sage. The lowest loss was found after freeze-drying. During freeze-drying, a low temperature is used, and contact with oxygen is limited, which results in reduced degradation of these compounds [44]. On the other hand, the greatest losses of carotenoids were measured in samples after drying at 30 °C. This could be due to easier oxidation of these components at higher temperatures in comparison to freeze-drying, as well as longer contact with oxygen compared to drying at higher temperatures (40 °C and 50 °C) [44]. Furthermore, thermal processes can damage some cellular structures, thereby releasing oxidative enzymes that decompose some antioxidant compounds such as carotenoids [45]. Generally, consumers store herbs at room temperature, and therefore the studied samples were stored under these conditions. A low temperature preserves quality, including the content of bioactive components [46]. On the other hand, storage at room temperature may cause loss of these compounds, as observed in our study. It is difficult to indicate the drying method that allows for the preservation of carotenoids during storage to the highest extent.

The content of total polyphenols in *Salvia hispanica* was 9.76 g·100 g^−1^ DW. To the best of our knowledge, there are no previous data on content of total polyphenolic compounds in the herb *Salvia hispanica*. Our results indicated that the herb *Salvia officinalis* contained 9.47 g·100 g^−1^ DW of total polyphenols. However, Jeshvaghani et al. [47] showed that *Salvia officinalis* leaves had 8.64 mg·100 g^−1^ DW of polyphenolic compounds, while Roby et al. [48] reported a value of 0.595 g·100 g^−1^ DW. Dent et al. [49], who studied ethanol and water extracts of sage from Croatia, also obtained lower values. The herb *Salvia officinalis* from Bulgaria, studied by Atanassova et al. [50], had 27.94 mg·100 g^−1^ DW of polyphenolic compounds. The level of total polyphenols in *Salvia sclarea* was 4.95 g·100 g^−1^ DW. Jeshvaghani et al. [47] obtained a higher value of 10.33 g·100 g^−1^ DW, while Taârit et al. [16] reported a lower level of polyphenols of 2.44 g·100 g^−1^ DW. Our results showed that the herb *Salvia hispanica* contained polyphenols at the same level as the herb *Salvia officinalis*. This suggests that the unknown *Salvia hispanica* herb, like the well-known *Salvia officinalis*, may have similar beneficial properties.

In all the tested species of sage, rosmarinic acid was the most abundant polyphenolic compound. The results of other studies also indicated that rosmarinic acid was the dominant polyphenolic compound in *Salvia officinalis.* Dent et al. [51] studied *Salvia officinalis* leaves from four wild field sites in the Mediterranean region of Croatia. Farhat et al. [52] analyzed *Salvia officinalis* from two different regions in north Tunisia at the vegetative, flowering, and fruiting stages. Hamrouni-Sellami et al. [53] evaluated *Salvia officinalis* from southern Tunisia, while Dent et al. [49] tested *Salvia officinalis* from the island of Pag (Croatia). These authors also detected phenolic acids such as p-hydroxybenzoic acid, caffeic acid, ferulic acid, gallic acid, p-coumaric acid, syringic acid, and vanillic acid, as well as flavonoids, mainly luteolin and apigenin (and their derivatives), hispidulin, and naringin. Additionally, Farhat et al. [52] and Hamrouni-Sellami et al. [53] identified the phenolic diterpenes carnosic acid and carnosol. Amato et al. [27], who for the first time showed a qualitative profile of the polyphenols in *Salvia hispanica* leaves, also detected compounds such as caffeic acid, chlorogenic acid, ferulic acid, rosmarinic acid, apigenin, luteolin, kaempferol, quercetin, and others. To the best of our knowledge, the polyphenol profile of the *Salvia sclarea* herb and the quantitative analysis of polyphenolic compounds in *Salvia hispanica* have not yet been evaluated.

The obtained results indicated that *Salvia hispanica* and *Salvia officinalis*, after freeze-drying, were characterized by the highest content of total polyphenols. Similar observations for *Salvia officinalis* were made by Sadowska et al. [31]. It is known that the low temperature of this process can protect polyphenolic compounds. Additionally, limited oxygen access can minimize the oxidation of these compounds [54]. Due to modification of the microstructure of freeze-dried plant tissues, the dried product is more porous, and thus the extraction process is more effective [55]. Furthermore, this process can inhibit the activity of some enzymes such as polyphenol oxidases, resulting in the protection of polyphenolic compounds [56]. Different polyphenol profiles in dried herbs, in comparison to fresh ones, can be due to the release of compounds after thermal treatment. The elevated temperature can influence the cell wall structure, promoting the release of internal compounds from the plant cells [57]. The greatest loss of polyphenols was found in samples of sage after drying at 50 °C, caused by the use of the highest temperature. Moreover, during natural drying (in comparison to freeze-drying) the degradation of polyphenol oxidases was not immediate; therefore, oxidation of these compounds may occur during the process [56]. The obtained results do not allow an indication of the drying method that protects polyphenols during storage to the highest extent.

Our research indicated that *Salvia hispanica* and *Salvia officinalis* were characterized by higher antioxidant activity compared to *Salvia sclarea*. These results were in agreement with the data reported by Jeshvaghani et al. [47], who studied various species of sage. However, Stagos et al. [6] indicated that the herb *Salvia sclarea* had the highest antioxidant activity among all the tested sage samples. The results of our study showed that the herb *Salvia hispanica* was characterized by a fairly high antioxidant activity.

## 5. Conclusions

The obtained results showed that the herb of a barely investigated species of sage, *Salvia hispanica*, was rich in polyphenolic compounds and showed high antioxidant activity. Both the polyphenol content and the antioxidant capacity were on the same level as those determined in the well-known *Salvia officinalis*, and were significantly higher compared to those found in *Salvia sclarea*. In all tested species of sage, rosmarinic acid was the most abundant polyphenolic compound. Furthermore, *Salvia hispanica* was rich in sinapinic acid, naringin, rutin, and carnosol. The use of different drying methods allowed us to indicate freeze-drying as the most effective for preserving polyphenols and carotenoids in the studied species of sage. Long-term storage up to 12 months resulted in a gradual reduction in antioxidant activity as well as in the content of polyphenols and carotenoids. However, even after such a long period of storage the herbs of all tested species of sage showed strong antioxidant activity and a high content of bioactive compounds. To conclude, the herb *Salvia hispanica* showed a high potential to be used as a rich source of bioactive compounds, including antioxidants, in the food industry, cosmetics, biomaterials, and active food packaging, similarly to the well-known *Salvia officinalis*.

## Figures and Tables

**Table 1:** Basic chemical composition of individual species of sage.

Species	Ash [g∙100 g^−1^ DW]	Protein [g∙100 g^−1^ DW]	Crude Fat [g∙100 g^−1^ DW]	Digestible Carbohydrates [g∙100 g^−1^ DW]	Dietary Fiber [g∙100 g^−1^ DW]
*Salvia hispanica*	11.73 ± 0.26 ^b^	9.43 ± 0.28 ^a^	1.67 ± 0.04 ^a^	30.26 ± 1.21 ^b^	46.90 ± 1.40 ^b^
*Salvia officinalis*	8.76 ± 0.25 ^a^	9.38 ± 0.06 ^a^	4.06 ± 0.06 ^c^	24.82 ± 2.41 ^a^	52.98 ± 2.10 ^c^
*Salvia sclarea*	18.39 ± 0.24 ^c^	14.49 ± 0.32 ^b^	1.99 ± 0.11 ^b^	28.17 ± 1.36 ^b^	36.96 ± 1.20 ^a^

Results are expressed as mean ± SD (*n* = 3). DW—dry weight. Mean values with different letters (a–c) within the individual columns are statistically different (*p* < 0.05).

**Table 2:** The content of bioactive compounds and antioxidant activity in fresh sage of individual species.

Species	Total Carotenoids [mg∙100 g^−1^ DW]	Total Polyphenols [g∙100 g^−1^ DW]	Antioxidant Activity [µmol Trolox∙g^−1^ DW]
*Salvia hispanica*	103.02 ± 0.10 ^b^	9.76 ± 0.52 ^b^	713.26 ± 36.72 ^b^
*Salvia officinalis*	63.48 ± 0.19 ^a^	9.47 ± 0.15 ^b^	651.48 ± 30.87 ^ab^
*Salvia sclarea*	109.06 ± 1.05 ^c^	4.95 ± 0.30 ^a^	568.49 ± 42.99 ^a^

Results are expressed as mean ± SD (*n* = 3). DW—dry weight. Mean values with different letters (a–c) within the individual columns are statistically different (*p* < 0.05).

**Table 3:** The content of total carotenoids in dried and stored sage of individual species.

Species	Drying Method	Storage							
		Directly after Drying		After 3 Months		After 6 Months		After 12 Months	
		Total Carotenoids [mg∙100 g^−1^ DW]	Changes * [%]	Total Carotenoids [mg∙100 g^−1^ DW]	Changes ** [%]	Total Carotenoids [mg∙100 g^−1^ DW]	Changes ** [%]	Total Carotenoids [mg∙100 g^−1^ DW]	Changes ** [%]
*Salvia hispanica*	freeze-drying	64.87 ± 0.68 ^l, D^	−37.03	54.90 ± 1.07 ^h, C^	−15.36	41.38 ± 0.26 ^h, B^	−36.21	29.24 ± 1.15 ^h, A^	−54.92
	natural drying	37.48 ± 0.91 ^f, D^	−63.62	24.76 ± 0.57 ^e, C^	−33.93	19.63 ± 0.30 ^d, B^	−47.63	13.94 ± 0.23 ^cd, A^	−62.81
	drying at 30 °C	27.93 ± 0.18 ^b, C^	−72.89	16.43 ± 0.37 ^a, B^	−41.16	14.26 ± 0.09 ^b, A^	−48.95	14.10 ± 0.09 ^de, A^	−49.50
	drying at 40 °C	29.41 ± 0.08 ^c, C^	−71.46	21.04 ± 0.07 ^c, B^	−28.45	21.10 ± 0.10 ^e, B^	−28.25	20.22 ± 0.09 ^g, A^	−31.25
	drying at 50 °C	33.79 ± 0.27 ^e, D^	−67.20	23.09 ± 0.22 ^d, C^	−31.67	18.73 ± 0.87 ^d, B^	−44.57	12.64 ± 0.42 ^c, A^	−62.60
*Salvia officinalis*	freeze-drying	57.98 ± 1.27 ^k, C^	−8.66	54.14 ± 0.08 ^h, B^	−6.63	41.82 ± 0.67 ^h, A^	−27.88	41.21 ± 1.00 ^j, A^	−28.93
	natural drying	45.85 ± 0.18 ^hi, C^	−27.77	39.51 ± 0.52 ^g, B^	−13.83	39.06 ± 0.93 ^g, B^	−14.80	35.17 ± 1.36 ^i, A^	−23.29
	drying at 30 °C	23.60 ± 0.08 ^a, C^	−62.82	19.80 ± 0.32 ^b, B^	−16.10	19.38 ± 0.28 ^d, AB^	−17.91	18.32 ± 0.65 ^f, A^	−22.38
	drying at 40 °C	31.44 ± 0.08 ^d, D^	−50.47	29.40 ± 0.95 ^f, C^	−6.47	24.82 ± 0.09 ^f, B^	−21.04	15.42 ± 0.41 ^e, A^	−50.97
	drying at 50 °C	31.79 ± 0.26 ^d, D^	−49.92	23.56 ± 0.17 ^d, C^	−25.89	14.60 ± 0.18 ^b, B^	−54.08	11.04 ± 0.13 ^b, A^	−65.28
*Salvia sclarea*	freeze-drying	71.04 ± 0.23 ^m, D^	−34.86	54.63 ± 1.11 ^h, C^	−23.10	49.60 ± 0.17 ^i, B^	−30.18	28.85 ± 0.19 ^h, A^	−59.38
	natural drying	46.68 ± 0.55 ^ij, D^	−57.20	23.62 ± 0.07 ^d, C^	−49.40	19.56 ± 0.09 ^d, B^	−58.10	14.78 ± 0.83 ^de, A^	−68.35
	drying at 30 °C	42.24 ± 0.78 ^g, D^	−61.27	20.98 ± 0.14 ^c, C^	−50.34	19.71 ± 0.18 ^d, B^	−53.35	17.56 ± 0.19 ^f, A^	−58.42
	drying at 40 °C	47.38 ± 0.53 ^j, C^	−56.56	22.41 ± 0.26 ^d, B^	−52.71	17.08 ± 0.53 ^c, A^	−63.95	16.91 ± 0.53 ^f, A^	−64.31
	drying at 50 °C	45.16 ± 1.42 ^h, D^	−58.59	23.39 ± 0.07 ^d, C^	−48.20	9.14 ± 0.00 ^a, B^	−79.77	5.85 ± 0.00 ^a, A^	−87.05

Results are expressed as mean ± SD (*n* = 3). DW—dry weight. Mean values with different letters (a–m) within the individual storage periods (columns) are statistically different (*p* < 0.05). Mean values with different letters (A–D) within the individual species of sage and drying methods (rows) are statistically different (*p* < 0.05). * Changes in the content of total carotenoids with reference to fresh sage samples. ** Changes in the content of total carotenoids with reference to sage samples directly after drying.

**Table 4:** The content of total polyphenols in dried and stored sage of individual species.

Species	Drying Method	Storage							
		Directly after Drying		After 3 Months		After 6 Months		After 12 Months	
		Total Polyphenols [g∙100 g^−1^ DW]	Changes * [%]	Total Polyphenols [g∙100 g^−1^ DW]	Changes ** [%]	Total Polyphenols [g∙100 g^−1^ DW]	Changes ** [%]	Total Polyphenols [g∙100 g^−1^ DW]	Changes ** [%]
*Salvia hispanica*	freeze-drying	11.84 ± 1.68 ^hi, A^	21.30	10.83 ± 0.58 ^i, A^	−8.46	10.40 ± 0.54 ^i, A^	−12.14	10.71 ± 0.24 ^f, A^	−9.49
	natural drying	10.79 ± 0.07 ^gh, B^	10.59	10.71 ± 0.24 ^hi, B^	−0.73	9.25 ± 0.12 ^gh, A^	−14.30	9.23 ± 0.47 ^e, A^	−14.44
	drying at 30 °C	9.73 ± 0.14 ^fg, C^	−0.28	9.16 ± 0.06 ^g, B^	−7.50	8.82 ± 0.10 ^g, A^	−10.99	8.57 ± 0.14 ^d, A^	−13.51
	drying at 40 °C	9.66 ± 0.02 ^f, D^	−0.97	9.25 ± 0.20 ^g, C^	−4.29	8.73 ± 0.05 ^g, B^	−9.68	8.34 ± 0.10 ^d, A^	−13.68
	drying at 50 °C	5.49 ± 0.15 ^a, C^	−43.78	4.71 ± 0.05 ^a, B^	−14.07	4.58 ± 0.01 ^a, B^	−16.55	4.09 ± 0.04 ^a, A^	−25.42
*Salvia officinalis*	freeze-drying	12.89 ± 0.63 ^i, B^	36.11	12.09 ± 0.25 ^j, AB^	−6.22	11.91 ± 0.50 ^j, AB^	−7.60	10.94 ± 0.69 ^f, A^	−15.14
	natural drying	12.03 ± 0.60 ^i, C^	27.02	10.09 ± 0.61 ^h, B^	−16.09	9.59 ± 0.38 ^h, B^	−20.25	8.17 ± 0.33 ^ds, A^	−32.11
	drying at 30 °C	7.90 ± 0.16 ^de, B^	−16.58	7.32 ± 0.54 ^de, AB^	−7.35	6.81 ± 0.00 ^f, A^	−13.74	6.64 ± 0.10 ^c, A^	−16.01
	drying at 40 °C	7.13 ± 0.05 ^bcd, C^	−24.72	7.15 ± 0.01 ^d, C^	0.27	6.19 ± 0.16 ^cde, B^	−13.23	5.59 ± 0.19 ^b, A^	−21.66
	drying at 50 °C	6.11 ± 0.07 ^ab, A^	−35.49	6.11 ± 0.47 ^bc, A^	0.07	5.95 ± 0.04 ^cd, A^	−2.66	5.94 ± 0.13 ^b, A^	−2.80
*Salvia sclarea*	freeze-drying	8.14 ± 0.01 ^de, C^	64.50	7.89 ± 0.15 ^ef, C^	−3.05	6.12 ± 0.21 ^cd, B^	−24.79	5.89 ± 0.33 ^b, A^	−27.63
	natural drying	8.52 ± 0.01 ^e, B^	72.15	8.04 ± 0.16 ^f, B^	−5.63	6.74 ± 0.01 ^ef, A^	−20.86	5.83 ± 0.42 ^b, A^	−31.55
	drying at 30 °C	7.69 ± 0.13 ^cde, B^	55.51	6.70 ± 0.16 ^cd, A^	−12.89	6.51 ± 0.50 ^def, A^	−15.33	5.92 ± 0.34 ^b, A^	−23.04
	drying at 40 °C	6.97 ± 0.11 ^bcd, D^	40.90	5.66 ± 0.04 ^b, C^	−18.83	5.14 ± 0.04 ^ab, B^	−26.27	4.49 ± 0.18 ^a, A^	−35.63
	drying at 50 °C	6.70 ± 0.27 ^bc, B^	35.45	6.33 ± 0.05 ^bc, B^	−5.53	5.70 ± 0.10 ^bc, A^	−14.99	5.74 ± 0.01 ^b, A^	−14.29

Results are expressed as mean ± SD (*n* = 3). DW—dry weight. Mean values with different letters (a–j) within the individual storage periods (columns) are statistically different (*p* < 0.05). Mean values with different letters (A–D) within the individual species of sage and drying methods (rows) are statistically different (*p* < 0.05). * Changes in the content of total polyphenols with reference to fresh sage samples. ** Changes in the content of total polyphenols with reference to sage samples directly after drying.

**Table 5:** Concentration of individual polyphenolic compounds in fresh, dried, and stored *Salvia hispanica*.

Polyphenolic Compounds	Fresh	Directly after Drying					After 12 Months of Storage				
		Freeze-Drying	Natural Drying	Drying at 30 °C	Drying at 40 °C	Drying at 50 °C	Freeze-Drying	Natural Drying	Drying at 30 °C	Drying at 40 °C	Drying at 50 °C
	mg∙100 g^−1^ DW										
**Phenolic Acids:**											
4-Hydroxybenzoic acid	nd	22.74 ± 0.22 ^h^	7.44 ± 0.08 ^c^	16.19 ± 0.02 ^f^	12.61 ± 0.00 ^e^	5.40 ± 0.00 ^b^	17.90 ± 0.11 ^g^	4.89 ± 0.10 ^a^	11.32 ± 0.21 ^d^	12.63 ± 0.19 ^e^	4.62 ± 0.04 ^a^
Caffeic acid	17.11 ± 0.06 ^h^	16.51 ± 0.06 ^g^	22.15 ± 0.00 ^i^	13.97 ± 0.00 ^f^	14.05 ± 0.06 ^f^	9.50 ± 0.00 ^c^	12.01 ± 0.23 ^e^	8.02 ± 0.04 ^b^	11.51 ± 0.06 ^d^	11.56 ± 0.00 ^d^	6.85 ± 0.00 ^a^
Chlorogenic acid	14.88 ± 0.00 ^e^	23.74 ± 0.16 ^j^	7.01 ± 0.04 ^c^	19.38 ± 0.08 ^g^	15.67 ± 0.02 ^f^	6.36 ± 0.00 ^a^	22.31 ± 0.32 ^i^	6.85 ± 0.50 ^bc^	20.92 ± 0.02 ^h^	10.00 ± 0.63 ^d^	6.95 ± 0.02 ^bc^
Ferulic acid	nd	11.25 ± 0.30 ^f^	3.92 ± 0.00 ^b^	11.64 ± 0.25 ^f^	6.51 ± 0.38 ^c^	2.39 ± 0.02 ^a^	7.28 ± 0.21 ^d^	2.79 ± 0.17 ^a^	7.79 ± 0.06 ^e^	6.53 ± 0.04 ^c^	2.47 ± 0.04 ^a^
Gallic acid	13.13 ± 0.00 ^c^	4.84 ± 0.04 ^b^	17.19 ± 0.16 ^d^	nd	nd	nd	3.66 ± 0.06 ^a^	3.64 ± 0.04 ^a^	nd	nd	nd
p-Coumaric acid	33.49 ± 0.06 ^c^	48.97 ± 0.04 ^k^	43.62 ± 0.00 ^j^	41.44 ± 0.04 ^i^	40.63 ± 0.06 ^h^	34.03 ± 0.02 ^e^	40.36 ± 0.00 ^g^	22.73 ± 0.03 ^a^	33.74 ± 0.04 ^d^	39.15 ± 0.06 ^f^	30.48 ± 0.02 ^b^
Rosmarinic acid	1358.80 ± 1.18 ^g^	1783.30 ± 0.91 ^k^	1769.02 ± 1.88 ^j^	1458.40 ± 1.10 ^h^	1315.27 ± 1.68 ^f^	200.31 ± 0.21 ^b^	1558.61 ± 1.04 ^i^	636.47 ± 1.63 ^c^	1139.64 ± 0.43 ^e^	905.93 ± 1.82 ^d^	170.86 ± 0.29 ^a^
Sinapinic acid	196.71 ± 0.00 ^h^	11.81 ± 0.65 ^f^	258.92 ± 0.16 ^i^	10.27 ± 0.04 ^e^	6.56 ± 0.00 ^c^	2.80 ± 0.10 ^a^	9.53 ± 0.02 ^d^	35.02 ± 0.03 ^g^	6.12 ± 0.19 ^bc^	5.77 ± 0.25 ^b^	2.80 ± 0.00 ^a^
Syringic acid	nd	11.48 ± 0.06 ^h^	5.71 ± 0.08 ^e^	8.79 ± 0.12 ^g^	4.60 ± 0.02 ^b^	4.95 ± 0.02 ^c^	5.51 ± 0.11 ^d^	3.10 ± 0.11 ^a^	6.68 ± 0.02 ^f^	4.74 ± 0.04 ^b^	4.60 ± 0.02 ^b^
Vanillic acid	5.78 ± 0.00 ^f^	5.30 ± 0.02 ^d^	7.50 ± 0.00 ^i^	6.14 ± 0.04 ^g^	6.53 ± 0.02 ^h^	5.42 ± 0.00 ^e^	4.33 ± 0.04 ^c^	3.88 ± 0.02 ^b^	6.18 ± 0.02 ^g^	6.44 ± 0.12 ^h^	3.45 ± 0.00 ^a^
**Flavonoids:**											
Apigenin	2.93 ± 0.06 ^h^	1.38 ± 0.02 ^g^	1.04 ± 0.00 ^e^	0.57 ± 0.02 ^d^	0.48 ± 0.04 ^c^	0.28 ± 0.02 ^b^	1.22 ± 0.04 ^f^	0.62 ± 0.04 ^d^	0.33 ± 0.02 ^b^	0.12 ± 0.00 ^a^	0.15 ± 0.00 ^a^
Epicatechin	nd	15.26 ± 1.30 ^d^	26.19 ± 0.82 ^g^	50.90 ± 0.70 ^j^	34.18 ± 1.31 ^i^	28.62 ± 0.29 ^h^	10.34 ± 0.32 ^a^	17.47 ± 0.90 ^e^	21.90 ± 0.06 ^f^	12.02 ± 0.50 ^ab^	12.89 ± 0.13 ^c^
Hesperidin	70.21 ± 0.87 ^a^	288.22 ± 0.40 ^h^	231.92 ± 7.51 ^g^	96.31 ± 2.30 ^d^	96.24 ± 1.58 ^d^	70.98 ± 1.17 ^a^	165.28 ± 10.76 ^f^	110.83 ± 8.20 ^e^	93.85 ± 2.87 ^cd^	84.09 ± 4.38 ^bc^	79.87 ± 0.87 ^ab^
Hispidulin	nd	14.24 ± 0.13 ^f^	nd	6.42 ± 0.02 ^c^	5.79 ± 0.06 ^a^	9.61 ± 0.04 ^d^	13.81 ± 0.02 ^e^	nd	6.06 ± 0.23 ^b^	5.99 ± 0.02 ^ab^	9.74 ± 0.04 ^d^
Isorhamnetin	15.80 ± 0.06 ^c^	0.99 ± 0.06 ^ab^	17.02 ± 1.39 ^d^	0.51 ± 0.04 ^a^	0.44 ± 0.00 ^a^	0.71 ± 0.04 ^a^	0.67 ± 0.02 ^a^	1.89 ± 0.22 ^b^	0.16 ± 0.02 ^a^	0.38 ± 0.04 ^a^	0.09 ± 0.00 ^a^
Kaempferol	nd	5.26 ± 0.00 ^h^	2.37 ± 0.24 ^b^	3.70 ± 0.00 ^e^	3.73 ± 0.04 ^e^	4.99 ± 0.04 ^g^	4.97 ± 0.02 ^g^	1.89 ± 0.01 ^a^	3.41 ± 0.06 ^d^	3.15 ± 0.02 ^c^	4.53 ± 0.00 ^f^
Luteolin	9.28 ± 0.37 ^e^	19.79 ± 0.13 ^j^	12.40 ± 0.41 ^f^	13.19 ± 0.08 ^g^	14.14 ± 0.04 ^h^	14.98 ± 0.08 ^i^	4.40 ± 0.08 ^c^	8.73 ± 0.01 ^d^	2.82 ± 0.16 ^a^	3.54 ± 0.10 ^b^	3.92 ± 0.08 ^b^
Myricetin	25.91 ± 0.00 ^b^	73.43 ± 0.14 ^h^	34.44 ± 0.08 ^c^	36.29 ± 0.00 ^d^	34.84 ± 1.13 ^c^	41.41 ± 0.10 ^f^	67.58 ± 0.04 ^g^	23.47 ± 0.05 ^a^	34.24 ± 0.21 ^c^	34.84 ± 0.02 ^c^	39.29 ± 0.11 ^e^
Naringin	199.07 ± 0.87 ^d^	309.70 ± 0.26 ^h^	294.69 ± 10.28 ^g^	223.92 ± 5.62 ^f^	225.10 ± 0.08 ^f^	212.95 ± 0.91 ^e^	291.40 ± 0.34 ^g^	143.23 ± 3.08 ^a^	173.93 ± 7.07 ^b^	175.57 ± 0.23 ^bc^	184.91 ± 0.04 ^c^
Quercetin	nd	4.10 ± 0.27 ^c^	8.83 ± 0.73 ^e^	6.43 ± 0.04 ^d^	9.82 ± 0.06 ^f^	4.79 ± 0.42 ^c^	2.18 ± 0.12 ^a^	4.17 ± 0.05 ^c^	3.32 ± 0.04 ^b^	5.98 ± 0.21 ^d^	4.31 ± 0.10 ^c^
Rutin	197.59 ± 0.74 ^c^	314.09 ± 1.73 ^g^	317.42 ± 3.59 ^g^	272.39 ± 4.98 ^e^	266.05 ± 1.66 ^e^	175.21 ± 1.93 ^b^	291.36 ± 4.33 ^f^	143.86 ± 4.57 ^a^	262.91 ± 6.59 ^e^	231.90 ± 10.99 ^d^	168.51 ± 0.42 ^b^
**Phenolic diterpenes:**											
Carnosic acid	17.90 ± 0.19 ^c^	34.29 ± 0.23 ^g^	53.42 ± 2.77 ^h^	20.92 ± 1.10 ^d^	29.26 ± 0.23 ^f^	16.37 ± 0.37 ^c^	24.63 ± 0.93 ^e^	10.03 ± 1.55 ^b^	7.92 ± 0.17 ^ab^	18.61 ± 1.01 ^cd^	5.69 ± 0.31 ^a^
Carnosol	128.60 ± 0.12 ^h^	47.82 ± 0.65 ^g^	41.31 ± 0.16 ^de^	44.30 ± 0.27 ^f^	42.11 ± 0.43 ^e^	40.12 ± 0.91 ^cd^	42.61 ± 0.49 ^e^	33.04 ± 1.11 ^b^	41.46 ± 0.65 ^e^	39.89 ± 0.12 ^c^	22.67 ± 0.04 ^a^

Results are expressed as mean ± SD (*n* = 3). DW—dry weight; nd—not determined. Mean values with different letters (a–k) within the individual rows are statistically different (*p* < 0.05).

**Table 6:** Concentration of individual polyphenolic compounds in fresh, dried, and stored *Salvia officinalis*.

Polyphenolic Compounds	Fresh	Directly after Drying					After 12 Months of Storage				
		Freeze-Drying	Natural Drying	Drying at 30 °C	Drying at 40 °C	Drying at 50 °C	Freeze-Drying	Natural Drying	Drying at 30 °C	Drying at 40 °C	Drying at 50 °C
	mg∙100 g^−1^ DW										
**Phenolic Acids:**											
4-Hydroxybenzoic acid	5.86 ± 0.12 ^ef^	31.01 ± 1.26 ^h^	30.09 ± 0.83 ^h^	6.28 ± 0.08 ^f^	4.02 ± 0.02 ^bcd^	4.98 ± 0.02 ^de^	10.22 ± 0.08 ^g^	4.71 ± 0.29 ^cd^	2.09 ± 0.02 ^a^	3.36 ± 0.00 ^b^	3.68 ± 0.04 ^bc^
Caffeic acid	33.97 ± 0.24 ^f^	80.07 ± 2.25 ^i^	45.83 ± 0.58 ^h^	36.79 ± 0.11 ^g^	24.53 ± 0.04 ^d^	23.72 ± 0.06 ^cd^	36.52 ± 0.19 ^g^	18.16 ± 0.02 ^a^	29.80 ± 0.02 ^e^	22.55 ± 0.04 ^bc^	21.5 ± 0.52 ^b^
Chlorogenic acid	44.23 ± 0.12 ^e^	62.72 ± 3.35 ^g^	56.84 ± 2.57 ^f^	15.04 ± 0.06 ^c^	11.68 ± 0.02 ^b^	12.55 ± 0.76 ^bc^	26.99 ± 0.04 ^d^	13.60 ± 0.49 ^bc^	12.08 ± 0.19 ^bc^	11.42 ± 0.04 ^b^	5.54 ± 0.06 ^a^
Ferulic acid	12.24 ± 0.00 ^a^	58.59 ± 0.15 ^h^	23.77 ± 0.33 ^d^	48.33 ± 0.04 ^g^	28.35 ± 0.10 ^e^	24.31 ± 0.16 ^d^	25.09 ± 1.28 ^d^	14.91 ± 1.00 ^b^	33.33 ± 0.14 ^f^	19.57 ± 0.00 ^c^	19.04 ± 1.04 ^c^
Gallic acid	11.47 ± 0.24 ^a^	63.88 ± 0.75 ^g^	16.74 ± 0.00 ^b^	30.06 ± 0.02 ^cd^	31.70 ± 0.06 ^d^	45.70 ± 2.69 ^e^	59.62 ± 0.04 ^f^	15.91 ± 0.14 ^b^	30.16 ± 0.44 ^cd^	29.25 ± 0.10 ^c^	44.64 ± 0.02 ^e^
p-Coumaric acid	4.83 ± 0.00 ^de^	5.22 ± 0.39 ^ef^	5.62 ± 0.50 ^f^	5.03 ± 0.02 ^de^	3.54 ± 0.08 ^c^	2.15 ± 0.02 ^a^	3.35 ± 0.21 ^bc^	2.05 ± 0.07 ^a^	4.69 ± 0.00 ^d^	2.94 ± 0.00 ^b^	2.13 ± 0.02 ^a^
Rosmarinic acid	1488.28 ± 17.50 ^f^	3479.39 ± 1.66 ^i^	2016.88 ± 28.89 ^h^	1341.99 ± 1.46 ^e^	1231.21 ± 0.78 ^d^	1167.06 ± 0.04 ^c^	1832.81 ± 77.86 ^g^	913.8 ± 0.55 ^a^	1177.83 ± 0.65 ^cd^	1078.66 ± 0.34 ^b^	1123.51 ± 15.71 ^bc^
Sinapinic acid	10.09 ± 0.00 ^d^	52.80 ± 0.11 ^h^	17.21 ± 1.16 ^f^	19.46 ± 0.10 ^g^	11.63 ± 0.10 ^e^	9.17 ± 0.10 ^d^	9.49 ± 0.56 ^d^	9.27 ± 0.31 ^d^	7.21 ± 0.02 ^c^	4.68 ± 0.12 ^b^	3.72 ± 0.02 ^a^
Syringic acid	19.66 ± 0.24 ^b^	49.23 ± 0.06 ^i^	49.40 ± 2.81 ^i^	30.83 ± 0.04 ^g^	25.82 ± 0.43 ^ef^	27.55 ± 0.65 ^f^	44.69 ± 1.34 ^h^	13.13 ± 0.09 ^a^	23.82 ± 0.04 ^de^	21.11 ± 0.02 ^bc^	23.10 ± 0.00 ^cd^
Vanillic acid	11.98 ± 0.00 ^d^	31.08 ± 0.17 ^h^	15.80 ± 0.66 ^e^	16.56 ± 0.15 ^f^	11.04 ± 0.06 ^c^	11.43 ± 0.04 ^c^	17.90 ± 0.08 ^g^	7.78 ± 0.05 ^a^	11.10 ± 0.06 ^c^	9.80 ± 0.02 ^b^	10.11 ± 0.23 ^b^
**Flavonoids:**											
Acacetin	26.98 ± 0.24 ^g^	0.77 ± 0.06 ^a^	1.05 ± 0.00 ^a^	10.17 ± 0.15 ^f^	8.29 ± 0.06 ^e^	5.49 ± 0.29 ^d^	nd	1.09 ± 0.15 ^a^	1.60 ± 0.08 ^b^	1.09 ± 0.02 ^a^	2.12 ± 0.00 ^c^
Apigenin	nd	0.22 ± 0.02 ^a^	nd	0.77 ± 0.00 ^c^	0.91 ± 0.02 ^d^	0.46 ± 0.00 ^b^	nd	0.78 ± 0.01 ^c^	nd	nd	nd
Catechin	nd	56.75 ± 3.47 ^f^	69.37 ± 2.40 ^g^	26.00 ± 0.48 ^d^	26.95 ± 0.02 ^d^	22.20 ± 1.10 ^c^	49.94 ± 0.36 ^e^	16.38 ± 1.92 ^a^	18.63 ± 0.02 ^ab^	17.24 ± 0.32 ^ab^	20.08 ± 0.18 ^bc^
Epicatechin	191.69 ± 2.87 ^e^	267.19 ± 13.52 ^f^	42.32 ± 1.41 ^d^	21.52 ± 0.4 ^abc^	17.49 ± 0.00 ^ab^	15.79 ± 0.06 ^a^	29.93 ± 0.29 ^c^	25.91 ± 0.04 ^bc^	17.34 ± 0.86 ^ab^	14.25 ± 0.66 ^a^	15.06 ± 0.59 ^a^
Hesperidin	346.43 ± 15.30 ^d^	23.97 ± 1.51 ^bc^	3.92 ± 0.25 ^a^	33.89 ± 0.33 ^c^	22.7 ± 0.08 ^b^	20.92 ± 0.21 ^b^	14.05 ± 0.06 ^ab^	3.55 ± 0.12 ^a^	14.75 ± 0.27 ^ab^	9.54 ± 0.10 ^a^	8.78 ± 0.04 ^a^
Hispidulin	nd	0.93 ± 0.02 ^a^	17.62 ± 0.75 ^c^	0.73 ± 0.02 ^a^	0.06 ± 0.00 ^a^	1.91 ± 0.00 ^b^	nd	nd	nd	nd	nd
Isorhamnetin	18.75 ± 0.91 ^d^	4.53 ± 0.21 ^c^	4.45 ± 0.33 ^c^	0.71 ± 0.02 ^ab^	0.68 ± 0.02 ^ab^	0.69 ± 0.02 ^ab^	0.96 ± 0.02 ^b^	nd	0.09 ± 0.00 ^a^	0.06 ± 0.00 ^a^	0.58 ± 0.04 ^ab^
Kaempferol	24.57 ± 1.83 ^c^	39.56 ± 0.04 ^d^	1.46 ± 0.08 ^a^	2.21 ± 0.02 ^ab^	2.08 ± 0.02 ^ab^	1.85 ± 0.04 ^a^	3.20 ± 0.23 ^b^	1.69 ± 0.03 ^a^	1.80 ± 0.02 ^a^	1.68 ± 0.08 ^a^	1.66 ± 0.02 ^a^
Luteolin	15.82 ± 0.79 ^e^	5.47 ± 0.25 ^c^	22.77 ± 0.41 ^f^	12.56 ± 0.08 ^d^	5.53 ± 0.06 ^c^	3.54 ± 0.02 ^ab^	5.24 ± 0.17 ^c^	5.12 ± 0.13 ^c^	3.91 ± 0.00 ^ab^	4.06 ± 0.02 ^b^	3.28 ± 0.19 ^a^
Myricetin	20.86 ± 0.24 ^h^	11.13 ± 0.06 ^g^	10.83 ± 0.25 ^f^	1.37 ± 0.04 ^c^	1.42 ± 0.04 ^c^	0.68 ± 0.02 ^b^	3.44 ± 0.10 ^e^	2.45 ± 0.18 ^d^	1.27 ± 0.08 ^c^	0.22 ± 0.02 ^a^	0.25 ± 0.02 ^a^
Naringin	523.85 ± 23.71 ^ef^	691.17 ± 2.68 ^h^	683.18 ± 47.10 ^h^	550.60 ± 1.22 ^f^	402.84 ± 0.41 ^cd^	440.54 ± 0.47 ^d^	596.81 ± 25.89 ^g^	304.06 ± 2.51 ^a^	494.46 ± 1.00 ^e^	350.50 ± 0.95 ^b^	396.56 ± 10.59 ^c^
Quercetin	9.74 ± 0.12 ^g^	10.92 ± 0.23 ^h^	10.95 ± 0.58 ^h^	8.70 ± 0.17 ^f^	7.95 ± 0.37 ^e^	7.09 ± 0.18 ^d^	9.18 ± 0.37 ^fg^	5.41 ± 0.09 ^c^	4.21 ± 0.06 ^b^	5.49 ± 0.02 ^c^	3.37 ± 0.23 ^a^
Rutin	317.77 ± 7.68 ^h^	27.01 ± 0.69 ^ef^	54.32 ± 4.80 ^g^	20.54 ± 0.19 ^cde^	17.55 ± 0.13 ^cd^	7.78 ± 0.65 ^ab^	24.40 ± 1.22 ^de^	33.30 ± 7.21 ^f^	15.22 ± 0.08 ^bc^	6.23 ± 0.10 ^a^	3.78 ± 0.08 ^a^
**Phenolic diterpenes:**											
Carnosic acid	22.63 ± 1.77 ^abc^	235.33 ± 1.40 ^gh^	249.48 ± 13.41 ^h^	225.95 ± 14.83 ^g^	196.02 ± 1.60 ^f^	132.17 ± 5.08 ^e^	36.51 ± 3.25 ^c^	98.80 ± 5.33 ^d^	26.63 ± 0.20 ^bc^	14.54 ± 0.50 ^ab^	10.52 ± 0.44 ^a^
Carnosol	151.86 ± 1.77 ^e^	381.79 ± 0.02 ^h^	133.87 ± 7.20 ^d^	273.13 ± 0.02 ^f^	302.41 ± 0.37 ^g^	275.11 ± 0.29 ^f^	27.84 ± 0.08 ^a^	120.38 ± 0.21 ^c^	39.20 ± 2.15 ^b^	40.71 ± 0.15 ^b^	37.80 ± 0.77 ^b^

Results are expressed as mean ± SD (*n* = 3). DW—dry weight; nd—not determined. Mean values with different letters (a–i) within the individual rows are statistically different (*p* < 0.05).

**Table 7:** Concentration of individual polyphenolic compounds in fresh, dried, and stored *Salvia sclarea*.

Polyphenolic Compounds	Fresh	Directly after Drying					After 12 Months of Storage				
		Freeze-Drying	Natural Drying	Drying at 30 °C	Drying at 40 °C	Drying at 50 °C	Freeze-Drying	Natural Drying	Drying at 30 °C	Drying at 40 °C	Drying at 50 °C
	mg∙100 g^−1^ DW										
**Phenolic Acids**											
4-Hydroxybenzoic acid	57.28 ± 0.17 ^k^	20.11 ± 0.12 ^j^	18.09 ± 0.00 ^i^	6.59 ± 0.11 ^e^	6.26 ± 0.02 ^d^	9.73 ± 0.04 ^h^	7.74 ± 0.04 ^f^	5.53 ± 0.00 ^c^	5.09 ± 0.04 ^a^	5.28 ± 0.00 ^b^	8.03 ± 0.02 ^g^
Caffeic acid	31.94 ± 0.00 ^i^	42.61 ± 0.00 ^k^	15.47 ± 0.00 ^e^	17.48 ± 0.04 ^g^	19.57 ± 0.02 ^h^	33.61 ± 0.02 ^j^	15.04 ± 0.00 ^d^	8.94 ± 0.03 ^a^	12.51 ± 0.00 ^c^	11.93 ± 0.00 ^b^	15.62 ± 0.10 ^f^
Chlorogenic acid	70.61 ± 0.34 ^i^	54.28 ± 0.08 ^h^	34.98 ± 0.17 ^f^	27.12 ± 0.23 ^d^	34.38 ± 0.04 ^e^	35.54 ± 0.02 ^g^	35.49 ± 0.04 ^g^	19.89 ± 0.04 ^c^	27.11 ± 0.02 ^d^	4.71 ± 0.19 ^b^	3.87 ± 0.08 ^a^
Ferulic acid	7.57 ± 0.51 ^a^	17.28 ± 0.76 ^f^	27.55 ± 0.25 ^h^	20.36 ± 0.02 ^g^	20.20 ± 0.48 ^g^	13.70 ± 0.00 ^c^	13.77 ± 0.00 ^c^	8.79 ± 0.14 ^b^	15.94 ± 0.08 ^e^	15.10 ± 0.04 ^d^	8.54 ± 0.00 ^b^
Gallic acid	40.71 ± 0.51 ^a^	699.97 ± 0.10 ^k^	293.84 ± 0.25 ^c^	453.90 ± 0.29 ^g^	421.89 ± 0.27 ^e^	604.09 ± 0.31 ^i^	648.01 ± 0.08 ^j^	252.22 ± 0.58 ^b^	424.33 ± 0.02 ^f^	416.35 ± 0.48 ^d^	563.42 ± 2.65 ^h^
p-Coumaric acid	nd	2.09 ± 0.02 ^d^	nd	1.86 ± 0.02 ^b^	2.16 ± 0.10 ^d^	2.07 ± 0.00 ^cd^	1.36 ± 0.02 ^a^	nd	1.94 ± 0.02 ^bc^	1.91 ± 0.02 ^b^	1.95 ± 0.10 ^bc^
Rosmarinic acid	346.32 ± 1.02 ^a^	2167.94 ± 0.23 ^i^	1839.50 ± 0.59 ^g^	1730.68 ± 0.33 ^g^	1891.31 ± 1.66 ^h^	2368.20 ± 1.49 ^j^	382.38 ± 0.41 ^b^	1101.51 ± 0.10 ^c^	1108.78 ± 0.76 ^d^	1203.75 ± 0.04 ^e^	1296.03 ± 0.64 ^f^
Sinapinic acid	20.65 ± 0.34 ^d^	25.81 ± 0.49 ^e^	10.23 ± 0.17 ^b^	18.09 ± 1.90 ^c^	20.93 ± 0.04 ^d^	29.88 ± 0.04 ^f^	3.74 ± 0.00 ^a^	9.41 ± 0.46 ^b^	3.33 ± 0.00 ^a^	3.24 ± 0.10 ^a^	18.13 ± 0.04 ^c^
Syringic acid	7.81 ± 0.17 ^a^	29.07 ± 0.02 ^h^	63.36 ± 0.08 ^i^	24.51 ± 0.02 ^g^	23.6 ± 0.00 ^f^	29.31 ± 0.00 ^h^	18.15 ± 0.04 ^c^	13.66 ± 0.01 ^b^	19.49 ± 0.02 ^d^	19.85 ± 0.33 ^e^	24.43 ± 0.02 ^g^
Vanillic acid	11.29 ± 0.34 ^g^	2.62 ± 0.06 ^cde^	3.81 ± 0.00 ^f^	2.70 ± 0.06 ^de^	2.82 ± 0.04 ^e^	2.51 ± 0.04 ^cd^	2.51 ± 0.04 ^cd^	1.59 ± 0.08 ^a^	2.42 ± 0.02 ^bc^	2.40 ± 0.04 ^bc^	2.17 ± 0.02 ^b^
**Flavonoids**											
Acacetin	nd	11.59 ± 0.04 ^g^	3.81 ± 0.00 ^e^	2.25 ± 0.00 ^c^	2.84 ± 0.02 ^d^	1.13 ± 0.04 ^b^	6.83 ± 0.02 ^f^	0.76 ± 0.01 ^a^	nd	nd	nd
Apigenin	6.72 ± 0.34 ^c^	nd	3.33 ± 0.17 ^b^	nd	nd	nd	nd	0.45 ± 0.03 ^a^	nd	nd	nd
Epicatechin	89.34 ± 0.34 ^d^	131.06 ± 0.04 ^g^	128.69 ± 0.25 ^f^	97.95 ± 0.31 ^e^	145.49 ± 0.27 ^h^	211.60 ± 0.31 ^j^	87.21 ± 0.60 ^c^	86.06 ± 0.07 ^b^	15.65 ± 0.02 ^a^	15.01 ± 0.17 ^a^	180.71 ± 0.55 ^i^
Hesperidin	33.02 ± 0.17 ^g^	122.87 ± 0.08 ^i^	131.6 ± 0.17 ^j^	161.50 ± 0.08 ^k^	11.28 ± 0.08 ^d^	85.56 ± 0.04 ^h^	7.95 ± 0.10 ^a^	25.76 ± 0.01 ^f^	18.57 ± 0.11 ^e^	8.43 ± 0.06 ^b^	10.83 ± 0.10 ^c^
Isorhamnetin	61.48 ± 1.70 ^e^	4.36 ± 0.04 ^bc^	30.46 ± 0.84 ^d^	3.67 ± 0.02 ^b^	4.11 ± 0.02 ^b^	3.92 ± 0.08 ^b^	3.34 ± 0.02 ^b^	5.43 ± 0.10 ^c^	0.39 ± 0.04 ^a^	0.62 ± 0.00 ^a^	0.55 ± 0.04 ^a^
Kaempferol	nd	9.33 ± 0.04 ^f^	nd	2.71 ± 0.04 ^a^	4.19 ± 0.04 ^d^	8.35 ± 0.02 ^e^	3.78 ± 0.02 ^c^	nd	3.24 ± 0.04 ^b^	nd	nd
Luteolin	31.7 ± 0.34 ^g^	5.41 ± 0.02 ^de^	17.67 ± 1.26 ^f^	4.62 ± 0.29 ^cd^	3.71 ± 0.08 ^bc^	5.81 ± 0.06 ^e^	4.08 ± 0.02 ^bc^	4.47 ± 0.14 ^cd^	2.72 ± 0.15 ^a^	2.29 ± 0.08 ^a^	3.15 ± 0.12 ^ab^
Myricetin	nd	31.33 ± 0.10 ^e^	11.66 ± 0.17 ^b^	37.63 ± 0.02 ^h^	36.22 ± 0.25 ^g^	12.25 ± 0.02 ^c^	28.46 ± 0.04 ^d^	1.22 ± 0.09 ^a^	32.32 ± 0.00 ^f^	nd	nd
Naringin	30.26 ± 1.70 ^a^	186.74 ± 0.19 ^f^	252.37 ± 1.68 ^i^	271.53 ± 1.53 ^j^	300.27 ± 0.17 ^k^	128.11 ± 0.08 ^d^	148.44 ± 0.31 ^e^	108.01 ± 0.15 ^b^	247.12 ± 0.14 ^h^	237.02 ± 0.19 ^g^	120.52 ± 0.04 ^c^
Quercetin	nd	3.12 ± 0.19 ^c^	nd	5.33 ± 0.14 ^e^	6.13 ± 0.06 ^f^	3.54 ± 0.14 ^d^	1.36 ± 0.02 ^a^	nd	2.90 ± 0.15 ^c^	3.43 ± 0.08 ^d^	1.98 ± 0.02 ^b^
Rutin	127.41 ± 0.85 ^e^	207.67 ± 0.11 ^g^	52.77 ± 2.27 ^d^	236.58 ± 0.04 ^h^	8.55 ± 0.02 ^b^	146.97 ± 0.04 ^f^	7.46 ± 0.14 ^b^	46.17 ± 0.17 ^c^	5.23 ± 0.13 ^b^	4.88 ± 0.06 ^a^	8.04 ± 0.16 ^b^
**Phenolic diterpenes**											
Carnosol	296.72 ± 10.70 ^f^	108.71 ± 4.33 ^de^	76.03 ± 0.67 ^b^	91.84 ± 0.00 ^c^	116.73 ± 0.23 ^e^	115.40 ± 6.26 ^de^	93.59 ± 0.27 ^c^	65.92 ± 4.48 ^a^	73.74 ± 0.25 ^ab^	107.78 ± 0.23 ^de^	106.56 ± 0.08 ^d^
Carnosic acid	53.44 ± 0.51 ^f^	113.04 ± 5.55 ^h^	56.46 ± 2.94 ^f^	76.75 ± 2.08 ^g^	46.33 ± 2.07 ^e^	35.25 ± 1.42 ^d^	35.55 ± 2.37 ^d^	1.53 ± 0.10 ^a^	9.16 ± 0.48 ^bc^	6.69 ± 0.44 ^b^	13.99 ± 0.57 ^c^

Results are expressed as mean ± SD (*n* = 3). DW—dry weight; nd—not determined. Mean values with different letters (a–k) within the individual rows are statistically different (*p* < 0.05).

**Table 8:** The antioxidant activity in dried and stored sage of individual species.

Species	Drying Method	Storage							
		Directly after Drying		After 3 Months		After 6 Months		After 12 Months	
		Antioxidant Capacity [µmol Trolox∙g^−1^ DW]	Changes * [%]	Antioxidant Capacity [µmol Trolox∙g^−1^ DW]	Changes ** [%]	Antioxidant Capacity [µmol Trolox∙g^−1^ DW]	Changes ** [%]	Antioxidant Capacity [µmol Trolox∙g^−1^ DW]	Changes ** [%]
*Salvia hispanica*	freeze-drying	1069.05 ± 33.52 ^h, C^	49.88	869.48 ± 34.27 ^hi, B^	−18.67	639.10 ± 10.29 ^h, A^	−40.22	621.54 ± 21.09 ^g, A^	−41.86
	natural drying	1074.27 ± 10.56 ^h, D^	50.61	897.15 ± 10.33 ^i, C^	−16.49	681.72 ± 5.16 ^i, B^	−36.54	614.79 ± 10.49 ^g, A^	−42.77
	drying at 30 °C	840.23 ± 10.67 ^e, B^	17.80	811.02 ± 7.92 ^h, B^	−3.48	520.08 ± 18.23 ^f, A^	−38.10	506.33 ± 8.00 ^f, A^	−39.74
	drying at 40 °C	849.28 ± 10.32 ^ef, C^	19.07	816.21 ± 44.56 ^h, C^	−3.89	522.63 ± 12.90 ^f, B^	−38.46	420.40 ± 2.65 ^e, A^	−50.50
	drying at 50 °C	507.94 ± 15.76 ^a, C^	−28.79	361.70 ± 2.60 ^a, B^	−28.79	125.97 ± 2.60 ^a, A^	−75.20	118.31 ± 7.79 ^a, A^	−76.71
*Salvia officinalis*	freeze-drying	903.76 ± 23.36 ^fg, C^	38.72	880.35 ± 23.52 ^i, BC^	−2.59	797.19 ± 36.59 ^j, B^	−11.79	696.57 ± 34.02 ^h, A^	−22.92
	natural drying	925.10 ± 68.11 ^g, B^	42.00	880.64 ± 57.63 ^i, B^	−4.81	837.41 ± 10.49 ^k, B^	−9.48	698.27 ± 13.12 ^h, A^	−24.52
	drying at 30 °C	761.31 ± 25.67 ^d, B^	16.86	728.49 ± 42.77 ^g, B^	−4.31	516.25 ± 2.57 ^f, A^	−32.19	491.67 ± 33.68 ^f, A^	−35.42
	drying at 40 °C	684.65 ± 20.86 ^c, D^	5.09	585.95 ± 10.38 ^ef, C^	−14.42	472.21 ± 5.19 ^de, B^	−31.03	379.18 ± 10.65 ^d, A^	−44.62
	drying at 50 °C	502.64 ± 28.70 ^a, B^	−22.85	421.65 ± 18.44 ^b, A^	−16.11	425.38 ± 18.44 ^bc, A^	−15.37	382.54 ± 21.07 ^d, A^	−23.89
*Salvia sclarea*	freeze-drying	623.16 ± 23.58 ^b, D^	9.62	579.74 ± 18.73 ^ef, C^	−6.97	504.06 ± 2.68 ^ef, B^	−19.11	321.65 ± 2.61 ^c, A^	−48.38
	natural drying	661.59 ± 21.30 ^bc, C^	16.38	627.05 ± 26.76 ^f, BC^	−5.22	582.52 ± 15.98 ^g, B^	−11.95	364.53 ± 7.87 ^d, A^	−44.90
	drying at 30 °C	606.23 ± 29.43 ^b, C^	6.64	552.12 ± 21.29 ^de, C^	−8.93	461.04 ± 34.12 ^cd, B^	−23.95	306.36 ± 13.34 ^c, A^	−49.46
	drying at 40 °C	544.32 ± 18.56 ^a, D^	−4.25	507.08 ± 7.96 ^cd, C^	−6.84	461.79 ± 5.32 ^cd, B^	−15.16	249.15 ± 2.66 ^b, A^	−54.23
	drying at 50 °C	500.07 ± 7.96 ^a, C^	−12.04	459.19 ± 10.50 ^bc, BC^	−8.18	420.82 ± 29.17 ^b, B^	−15.85	227.92 ± 15.67 ^b, A^	−54.42

Results are expressed as mean ± SD (*n* = 3). DW—dry weight. Mean values with different letters (a–k) within the individual storage periods (columns) are statistically different (*p* < 0.05). Mean values with different letters (A–D) within the individual species of sage and drying methods (rows) are statistically different (*p* < 0.05). * Changes in the antioxidant activity with reference to fresh sage samples. ** Changes in the antioxidant activity with reference to sage samples directly after drying.

## Data Availability

The data presented in this study are available on request from the corresponding author.

## Sample Availability

Samples of the compounds are not available from the authors.
